# Effects of Endocrine Disrupting Chemicals (EDCs) on Skeletal System Development: A Review

**DOI:** 10.7759/cureus.46109

**Published:** 2023-09-28

**Authors:** Chanemougavally J, Balaji Thotakura, Shruthy K M., Janaki C. S.

**Affiliations:** 1 Anatomy, A.C.S Medical College and Hospital, Dr. M.G.R Educational and Research Institute, Chennai, IND; 2 Anatomy, Chettinad Hospital and Research Institute, Chettinad Academy of Research and Education, Chennai, IND; 3 Anatomy, Bhaarath Medical College and Hospital, Chennai, IND

**Keywords:** estrogen, skeletal system malformation, congenital anomaly, bpa, edc

## Abstract

Introduction: Endocrine-disrupting chemicals (EDCs) are exogenous substances that alter endocrine function and cause adverse effects on an organism. EDC interference with the endocrine system leads to chronic autoimmune disorders, abnormal osteogenesis, infertility, and reproductive, neurological, cardiovascular, and metabolic disorders. Among the adverse effects of EDCs are their impact on developing fetuses and neonates. EDCs like bisphenol A (BPA), pesticides, and lead interfere with or alter sex steroid hormone synthesis and metabolism, leading to developmental delay, infertility, and urogenital carcinoma in both sexes.

Objective: This review article examines the most harmful EDC, BPA, which affects the skeletal system during the embryonic period. The literature investigates the effects of BPA on various systems in our body, but the mechanism behind skeletal system development during the embryonic period is still unknown.

Materials and methods: In the present review, 25 articles were reviewed through multiple windows like PubMed, Scopus, and Web of Science. Many articles mention the effects of BPA on the skeletal system after birth and also examine reproductive system abnormalities, hereditary characteristics, excretory system malfunctions, and physical and mental illness in various mechanisms.

Discussion: The impact of BPA on the skeletal system causes morphological and physiological changes in developing embryos. The general ideology regarding skeletal system development and its mechanism is as follows: the formation of bone (osteocytes) is reduced by the apoptosis of precursor bone cells (osteoblasts) by the effect of BPA.

Conclusion: EDC exposure induces the apoptosis of bone cells and inhibits the formation of osteoblasts, and long-term exposure to these chemicals will also impact immune system development.

## Introduction and background

Endocrine-disrupting chemicals (EDCs) are exogenous substances that are involved in the alternation of synthesis and the secretion and metabolism of natural hormones. Natural hormones like testosterone, parathormone, etc. present in the body are meant for growth, reproduction, and homeostasis. EDCs exert their actions mainly by disrupting normal hormone receptors like estrogen receptors, androgen receptors, progesterone receptors, and thyroid receptors [1]. Globally, there are more than 1,000 chemicals with endocrine-acting properties. EDCs have become a global problem for the environment and human health. EDCs are present in regularly used materials and substances like pesticides including insecticides, industrial chemicals, metals, pharmaceutical agents, and plasticizers. Human beings are exposed to EDCs in their day-to-day lives either by ingestion, inhalation, or to some extent, dermal uptake. Many EDCs are bioaccumulates, deposited in fat tissue. Identifying EDCs in the human body is difficult because the adverse effects develop later in old age [2]. The highest fatal adverse effects of EDCs are their impacts on developing fetuses and neonates. EDCs interfere with or alter sex steroid hormone synthesis and metabolism, thus leading to developmental delay, infertility, and urogenital carcinoma in both genders [2]. Some of the EDCs affect the skeletal system development of fetuses and neonates.

EDCs

EDCs mimic estrogen hormones and disrupt the normal mechanism of our complex endocrine system [3]. An EDC has been defined by WHO (2002) in the International Programme on Chemical Safety as “an exogenous substance or compound that interferes with the function of the endocrine system and causes unwanted effects in an intact organism or its population” [4].

Frequent exposure to EDCs causes adverse effects during development because the endocrine system exhibits cell, tissue, and receptor-specific actions during the life cycle, and EDCs can produce complex effects [5]. The interference of EDCs with the endocrine system leads to chronic autoimmune disorders, abnormal osteogenesis, infertility [4], and reproductive, neurological, cardiovascular, and metabolic disorders [6]. In younger children, EDC exposure causes a high risk of obesity [7]. EDCs promote abnormal skeletal development via various disruptive pathways [8].

Table 1 shows the different EDCs and the developmental diseases they induce.

**Table 1:** EDC chemicals with abnormalities EDC: endocrine-disrupting chemical, BPA: bisphenol A, PCBs: polychlorinated biphenyls

S. No.	EDC substance	System involved	Abnormalities
1	BPA, PCBs, estrogens, pesticides, and phthalates	Reproductive and endocrine	Carcinoma of the breast, infertility, diabetes, early puberty
2	BPA and PCBs	Respiratory and cardiovascular	Asthma, stroke
3	PCBs, lead, and ethanol	Nervous	Alzheimer’s disease, hearing disabilities
4	Dioxin	Immune	Autoimmune disease and susceptibility to infection

Common endocrine disruptors

Several endocrine disruptors act like endocrine hormones that cause adverse effects on human health. Among them are the following:

Bisphenol A (BPA) is an industrial chemical that mimics estrogen; it is also called fake estrogen. BPA is commonly found in all forms of plastics such as sports water bottles, baby pacifiers, canned food containers, and polycarbonate plastics. It causes heart disease, obesity, infertility, and carcinoma of the prostate and breast. Recent researchers have determined that BPA can be detected in the bodies of 93% of humans globally.

Perchlorate is a combination of chloride and oxygen. Perchlorate can be found in highly processed food such as salami, plain bagels, and boxed mac and cheese. Some fresh, raw vegetables like broccoli, collard greens, and cauliflower test high for perchlorate. It causes great damage to metabolism and brain function.

Dioxins are toxic chemicals produced from burnt waste resulting from manufacturing chemicals and pesticides. Dioxins are not like other EDCs because once they are consumed, they stay in the human body for years, and cause damage to various organs.

Atrazine is an herbicide sprayed heavily on crops such as sugarcane, corn, wheat, and sorghum to stop broadleaf weeds. Pregnant women exposed to atrazine have a higher incidence of congenital anomalies in their newborns.

Phthalates are EDCs used to create solvents and soft plastics. They are commonly found in cosmetics, plastic wraps, and medical tubing. Phthalates disrupt the endocrine system leading to congenital birth defects, premature puberty in girls, and insulin resistance.

Chlorine is a toxic green gas found in laundry detergent, corporation water supplies, swimming pools, hot tubs, paper products, dyes, insecticides, and paint. Minimal exposure causes irritation to the skin, nausea, and persistent cough. A greater exposure time to chlorine leads to a decrease in androgens and thyroid function, cardiac failure, or lung edema. Chlorine was used as a weapon in World War I.

Lead is a heavy metal commonly used in paints and pipes and is commonly present in chili powder from Asian countries; moreover, 99% of lead has been found in kohl (eyeliner) and costume jewelry. Lead causes damage to the endocrine system along with brain damage, abortion, lower IQs, and developmental delay. It decreases the secretion of sex hormones.

Mercury is a toxic metal generally found in underground water. Mercury is found in batteries, fluorescent light bulbs, and seafood like mackerel, shark, tilefish, tuna, lobster, cod, and snappers. Ingestion of mercury during pregnancy leads to brain damage and developmental delay, and it disrupts the adrenalin, insulin, estrogen, and testosterone functions in the body leading to irregular menstruation and defective ovulation in progeny.

Perfluorinated chemicals (PFCs) are usually applied to repel stains on carpets and upholstery and prevent cooking residue on utensils. PFCs are used in carpets labeled as “stain resistant” and on coated cooking utensils. Researchers have determined that 99% of people globally have perfluorinated chemicals in their bodies. PFC exposure increases the risk of thyroid, liver, testes, and ovarian dysfunction because of the involvement of the endocrine system.

Glycol ether can be found in paint, oven cleaners, liquid soaps, perfumes, inks, and cosmetic products. More exposure to glycol ether will cause infertility, oligospermia, teratozoospermia, testicular agenesis, and abortion. Sudden exposure to glycol ether may cause extreme liver and kidney damage, pulmonary edema, or anemia, and it decreases bone marrow production and causes stress and autoimmune disorders [3].

## Review

Materials and methods

Search Methods for Identifying Studies

**Figure 1:**
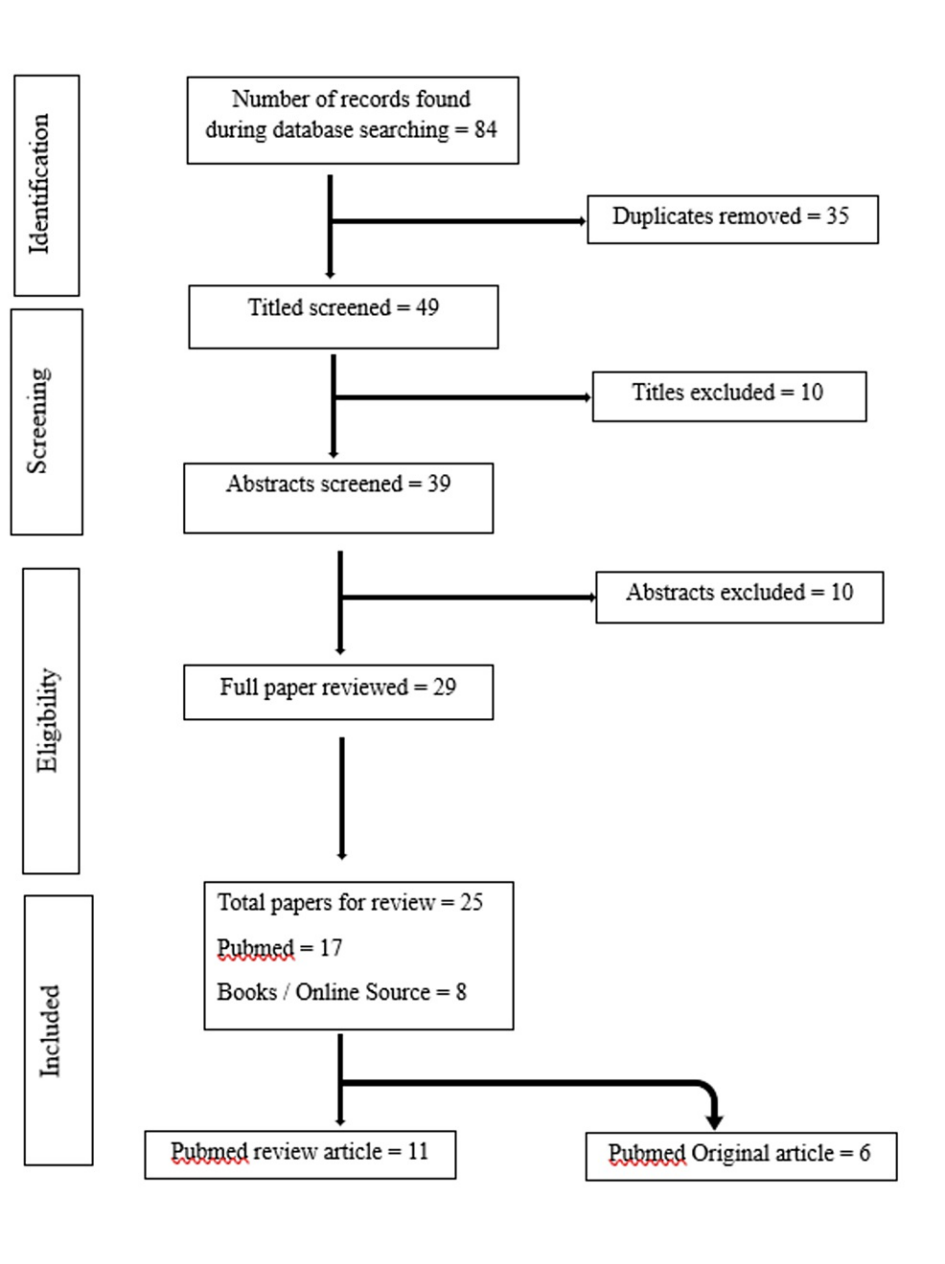
PRISMA flowchart for literature review

The articles were searched for in multiple windows of health sciences including PubMed, Scopus, and Web of Science. Most of the articles were retrieved from PUBMED. Articles published between 2003 to 2022 were included in the review. Many articles were reviewed: six were original studies and 11 were review articles. Keywords used were BPA OR bone OR bone homeostasis OR endocrine disruptors OR metabolism and mechanism of bone formation OR osteoblast lineage OR rank L signaling OR osteoporosis OR osterix PR xenoestrogen OR skeletal development OR regulation of bone. Figure 1 shows the Preferred Reporting Items for Systematic Reviews and Meta-Analyses (PRISMA) flowchart for the literature review.

Results

Out of 20 published articles included in our study, seven were from the USA, three were from the UK, and one article each was from Greece, Russia, and Nigeria. The sample groups included were mice, Wistar rats, women, and children. Most of the studies were human studies related to the reproductive system or endocrine system. Seven were animal studies and five were human-involved studies included in this review. Many review papers were related to human health care and endocrine disruption. Several review articles provided an overview of how BPA affects the mechanisms that disrupt human bone health. However, studies about skeletal system development with a BPA-injected model were either minimal or still unknown. Only seven studies analyzed the skeletal system, and many were review articles.

Overview of Skeletal System Development

After notochordal formation in the third week of intraembryonic life, the skeletal system begins to develop. Limb buds start to develop during the fourth week. During the fifth week of development, the limb begins to extend. At the end of the fifth week, the embryo has doubled its size. In the seventh week, the embryo enlarges, and facial features start to develop and become visible. At the end of the eighth week, the limb begins to extend and take on a distinct shape [8].

Skeletal system development plays a major role in the production of bone cells in bone growth, maintenance, and the stability and repair of bone tissue during injury. Condensation of mesenchymal cells differentiates into chondroblasts and osteoblasts, which later convert into cartilage and bone, respectively [9,10]. There are three types of cell lineage groups that play major roles in the development of the whole skeletal system in the human body [11]: osteoblasts (secrete hydroxyapatite crystals and calcium to increase mineralization to produce a new set of cells called osteocytes), osteocytes (mature bone cells), and osteoclasts (hematopoietic in origin) [12].

Osteoblasts are the precursor cells to bone formation. They secrete the extracellular matrix protein osteopontin [12], a cellular protein that facilitates normal physiological processes [9]. Deposition of calcium in the hydroxyapatite crystal with type I collagen facilitates structural support of the skeleton. Several osteoblasts join to form a unit of bone called the osteon (or the Haversian system) [12]. Some groups of osteoblasts undergo apoptosis, and another subset of osteoblasts is embedded into the bone matrix forming osteocytes. These osteocytes are responsible for mechanical and hormonal signals that control bone remodeling [12].

Osteoclasts

Osteoclasts are large multilayered cells involved in bone remodeling. These cells are formed from hematopoietic stem cells and transform into mature osteoclasts through the interaction of macrophage colony-stimulating factor (M-CSF) [12] and receptor activator of nuclear factor kβ ligand (RANKL). M-CSF facilitates the proliferation of osteoclast precursors, and RANKL initiates the transformation of undifferentiated osteoclast precursors to mature osteoclasts [12].

Mechanism of Skeletal System Development

EDCs alter the remodeling process of bones and the development of the skeletal system by hormonal regulation [13,14]. EDCs promote abnormal skeletal development by disrupting various pathways such as Wnt signaling and the beta-catenin pathway either directly or indirectly [15-17]. Osteocalcin, a non-collagenous protein in bone, induces bone formation, improves glucose metabolism and testosterone synthesis, and maintains muscle mass [18]. Alkaline phosphatase, a regulatory substance of bone mineralization, hydrolyses inorganic pyrophosphate for the synthesis of hydroxyapatite [19]. Osteoblast development is controlled by the transcription factors RUNX2 and Osterix; it requires Wnt signaling [20]. RUNX2 is a regulatory gene that produces a protein that is involved in the development and maintenance of teeth, bone, and cartilage. RUNX2 genes induce early pro-osteoblast differentiation and prevent mesenchymal cells from forming another cell [20].

Catenin signaling and the Wnt signaling pathway play a major role in skeletal development and the homeostasis of human health and disease [16]. The Wnt signaling pathway enhances osteoblast maturity and activity [15]. Osterix, also known as transcription factor SP7, is a protein in the SP7 gene that plays a major role along with RUNX2 in the differentiation of mesenchymal cells into osteoblasts and eventually osteocytes [17]. Terminal differentiation of osteoblasts requires the transcription factor AtF4 [20].

Osteoclast development depends on osteoblast cells. Osteoclast development requires a range of cytokines, steroids, and lipids, which are precursors [20]. Osteoblast expression enhances the formation and survival of osteoclast precursors and also regulates the RANK receptor. Osteoblasts express RANKL, which binds to the precursors of the RANK receptor on osteoblasts and their precursors. These interactions induce the proliferation of osteoclasts. Inactivation of RANK or RANKL leads to the complete absence of osteoclasts. A RANKL decoy receptor, osteoprotegerin, is secreted by the extracellular matrix of osteoblasts. It has a negative feedback mechanism between the osteoblast and osteoclast formation. Osteoprotegerin binds with RANKL, which prevents RANK from binding on osteoclast cells [20].

Discussion

Effect of EDC on Skeletal System Development

**Figure 2:**
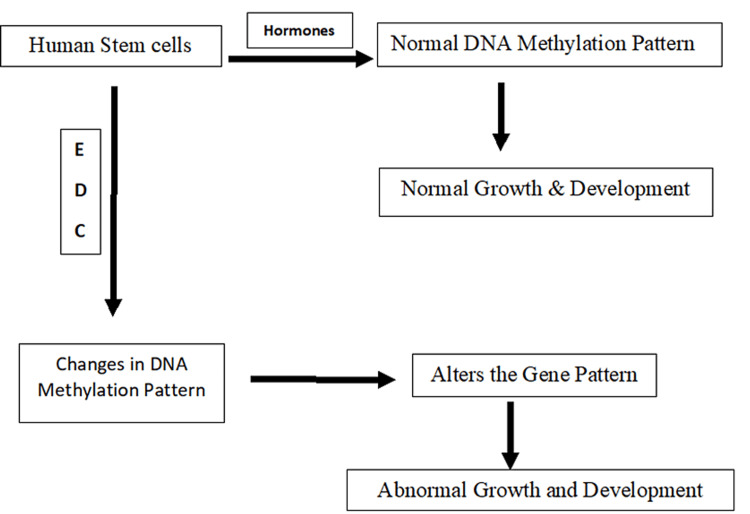
Mechanism of EDC on growth hormone EDC: endocrine-disrupting chemical Self-made figure

The bone microenvironment is a combination of osteoclasts and osteoblasts and is altered by numerous factors such as hormones and also through mediators of bone-forming cells secreted in response to normal physiological and pathological conditions. Humans are exposed to environmental estrogens at low doses. They alter the remodeling process of bones and the development of the skeletal system by their hormonal regulation [13]. The epigenetic mechanism is affected by epigenetic factors. Some of the processes are development and childhood, environmental chemicals, drugs and pharmaceuticals, and aging and diet. DNA methylation is a biological process by which methyl groups are added to the DNA molecule. Methylation can change the activity of a DNA segment without changing the sequence (Figure 2). These methyl groups are present in our diet and can activate or repress the genes in the target DNA.

Another mechanism is called histone modification. Histone-like proteins bind with DNA for compaction and gene regulation. The epigenetic factors bind to the histone tails and alter the DNA and availability of genes in DNA activation. Both DNA methylation and histone modification alter gene regulation with epigenetic factors. These processes cause cancer, autoimmune diseases, mental disorders, and diabetes.

**Figure 3:**
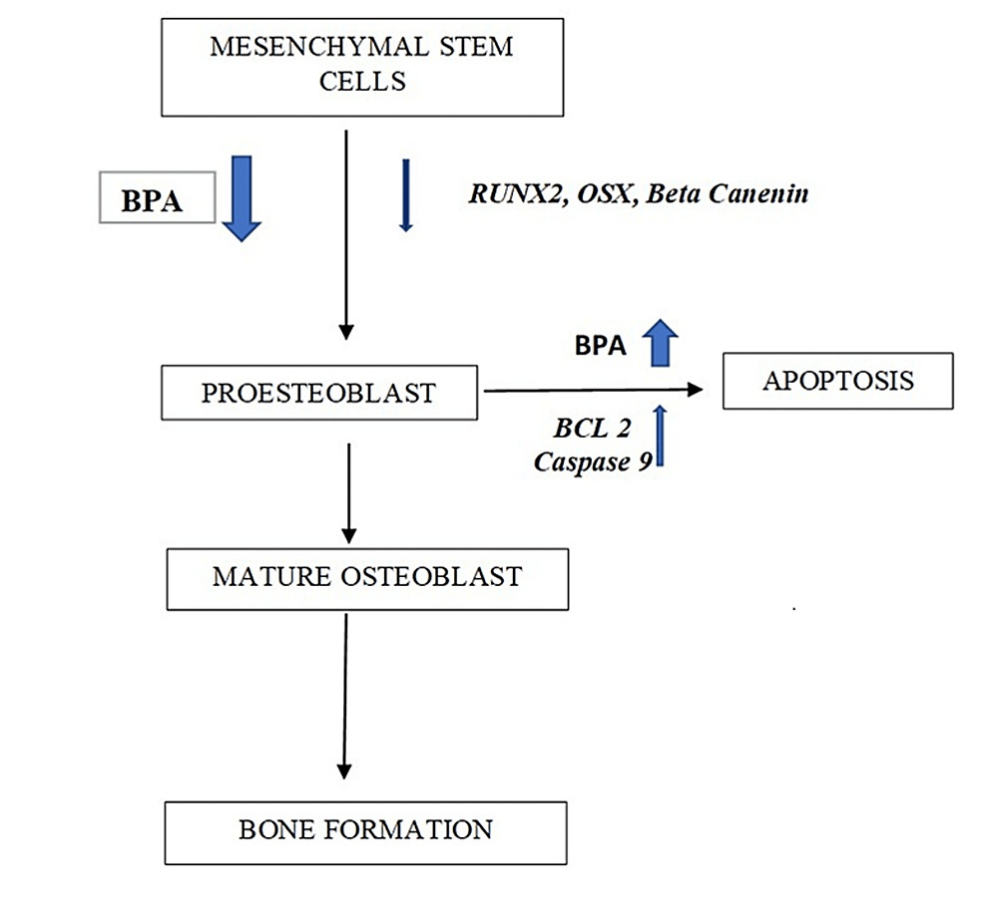
Effect of BPA on skeletal system development BPA: bisphenol A Self-made figure

In vitro studies show that BPA treatment induces osteoblast reduction and formation of bone cells via MC3T3-E1 preosteoblasts, alkaline phosphatase activity, and calcium nodule formation. BPA decreases the macrophages’ viability and the expression of BCL2 and caspase 3 and 8 (apoptotic initiators) [21]. The osteogenic activities of BPA on some responsive osteogenic tissues such as the walls of the uterus and uterine epithelium are not significant [22]. Osteoblast formation decreases the expression of genes such as RUNX2, osterix, and beta-catenin. Increased BCL2 gene expression (a pro-apoptotic gene) and caspase 9 (an apoptotic initiator) have been seen in the MC3t3-E1 apoptosis association. BPA increases the activity of alkaline phosphatase and the cellular calcium content. Bisphenol AF enriches the TGF-beta signaling pathway, whereas BPS reduces gene expression related to the Wnt signaling pathway and specific osteoblast markers (RUNX2 and osteoprotegerin; Figure 3) [23].

Damaged bone is reabsorbed by osteoclasts and induces the formation of new bone. Damaged bone heals because of excessive reabsorption. Overall in vitro studies of the effect of BPA on osteoclasts are limited [21].

Evidence from animal studies on the effect of BPA on the rodent skeletal system ranges from fetal to neonatal development of the skeletal system. In ovariectomized rats, BPA decreases tibial trabecular metaphyseal density in bone health. The osteocalcin level increases in BPA-treated rats and acts as a bone formation marker. Osteoblast-related gene expression is not suppressed by BADGE (BPA diglycidyl ether). A reduction of the morphology of bone health and trabecular thickness demonstrates that animals were receiving BADGE on bone structural indices (Table 2) [22].

**Table 2:** Study characteristics of included studies EDCs: endocrine-disrupting chemicals, BPA: bisphenol A

Author and year	Study design and country	Subject/age	Health impact	Results	Conclusion
Liu et al. 2022 [15]	Japan	Male and female Wistar rats	EDCs or any drug administration affect the Wnt signaling pathway in the early period of the fetus.	Abnormal activity of Wnt signaling induces oncogenic tumor formation and promotes various diseases.	Abnormal activity of Wnt signaling inhibits clinical problems like tumor metastasis and chemoresistance.
Bayram et al. 2020 [2]		Human	EDC interferes with the synthesis, action, and metabolism of sex steroids and can cause infertility, developmental problems, and hormone-sensitive cancer.	EDCs exhibit obesogenic effects that disturb energy homeostasis. Interference with the neuroendocrine axis has also been reported.	EDC affects the metabolism, action, and synthesis of hormones, bone health, and the immune system.
Lite et al. 2019 [24]	USA	Pregnant Wistar rats	BPA exposure during the postnatal period affects the weight of pups at birth and reduces anogenital distance.	BPA-exposed animals showed an increase in serum estradiol levels and a reduction in follicle‐stimulating hormone.	Long‐term prenatal BPA exposure affects postnatal ovarian health and function mediated by the altered miRNA expression.
Xin et al. [14]	France	Male and female mice	BPA acts like an estrogen, and it affects bone morphology and the mechanism of bone growth.	By DNA methylation and histone modification, BPA modifies skeletal system development and immune system dysfunction.	EDCs promote abnormal skeletal development by various disrupting pathways like Wnt signaling and the beta-catenin pathway either directly or indirectly. Abnormal Skeletal development affects physiological changes.
Lobstein et al. 2018 [7]	USA	Young adult	Endocrine disruptors cause obesity by multiple mechanisms.	EDCs create an imbalance between caloric intake and calories burnt especially by altering appetite, satiety, and food preferences.	In young children, a higher risk of obesity is observed when exposed to such hormone-disrupting chemicals.
Agas et al. 2012 [13]	Italy	Humans	EDCs alter the bone-remodeling process by the hormone regulation mechanism and abnormal skeletal formation.	Exposure to chemical compounds influences bone formation by osteoblast and osteoclast cells.	EDCs effect on mammalian bone provides the development of a macromolecular view of their action.
Schug et al. 2011 [6]	USA	Human adults and children	EDCs cause fetal-based adult diseases and disease susceptibility is also very high.	EDC exposure during the early stages of fetal development can impact the development of the central nervous system, head, limbs, skeleton, and reproductive system.	Prenatal or exposure to EDCs at an earlier age has a strong association with increased incidence of carcinoma of the breast and prostate. Parkinson's disease, obesity, and other diseases
Diamanti-Kandarakis et al. 2009 [1]	Athens	Mice	Exposure to BPA during the prenatal period and neoplasia of mammary glands in rat models resembles estrogen dependency and histopathology.	Exposure to estrogens throughout a woman’s life, including the period of intrauterine development, is a risk factor for the development of breast cancer.	EDCs alter the morphogenesis of mammary glands and abnormal development of mammary glands, more prone to neoplastic development.

BPA interferes with human fetal and adult bone cell formation via the steroid receptor, modifying the rate of cell proliferation and accumulation of collagen in osteoblasts. Previous studies have reported a reduction in the length and trabecular area of the femur in male rats exposed to low-dose BPA concentrations in their drinking water. High-dose exposure to BPA caused non-significant tendencies toward an increase in the number of trabeculae and the amount of bone volume fraction and bone marrow area in female rats [22]. EDCs can have harmful effects on bone modeling and remodeling through paracrine hormone synthesis and the release of systemic hormones [23]. Long‐term prenatal BPA exposure induces effects on postnatal ovarian health and function mediated by altered miRNA expression [24]. Endocrine disorders are the fifth principal cause of mortality worldwide. In recent years, miRNA’s regulatory mechanism could be more effective in diagnostic and therapeutic tools for treating people who have various biological processes associated with endocrine disorders [25].

## Conclusions

EDC exposure induces the apoptosis of bone cells and inhibits the formation of osteoblasts. RUNX2 and Osterix inhibit the differentiation of osteoblasts at the preosteoblast level. Osteoclast formation depends on osteoblast formation. Because the Wnt signaling pathway is interrupted, osteoclast remodeling initiates skeletal dysfunction and morphological changes.

Many studies have been conducted on reproductive system anomalies in both male and female populations following EDC exposure. Different dosage levels have been used for the analysis of EDC toxicity and human exposure. Endocrine system dysfunction causes physical and mental imbalances for the individual and their offspring. Long-term exposure to these chemicals will also impact immune system development. This review analyzed the effects of EDCs on skeletal system development especially during postnatal life. Extensive research is required to identify the exact mechanism of congenital malformation during skeletal system development in the embryo.
